# An interactive course program on nutrition for medical students: interdisciplinary development and mixed-methods evaluation

**DOI:** 10.1186/s12909-024-06596-4

**Published:** 2025-01-23

**Authors:** Gonza B. Ngoumou, Daniela A. Koppold, Laetitia Wenzel, Anne Schirmaier, Carolin Breinlinger, Lisa M. Pörtner, Stefan Jordan, Julia K. Schiele, Etienne Hanslian, Annika Koppold, Beate Stock-Schröer, Dimitra M. Varvarezou, Michael Jeitler, Miriam Ortiz, Andreas Michalsen, Wiebke Stritter, Georg Seifert, Christian S. Kessler

**Affiliations:** 1 Charité Competence Center for Traditional and Integrative Medicine (CCCTIM), Charité – Universitaetsmedizin Berlin, Corporate Member of Freie Universitaet Berlin, Humboldt-Universitaet zu Berlin and Berlin Institute of Health, Berlin, Germany; 2 Department of Internal Medicine and Nature-Based Therapies, Immanuel Hospital Berlin, Berlin, Germany; 3 Institute of Social Medicine, Epidemiology and Health Economics, Charité-Universitaetsmedizin Berlin, Corporate Member of Freie Universitaet Berlin and Humbolt-Universitaet zu Berlin, Berlin, Germany; 4 Department for Prevention and Care of Diabetes, Department of Medicine III, Faculty of Medicine Carl Gustav Carus, Technische Universitaet Dresden, 01307 Dresden, Germany; 5 Faculty of Medicine, Otto-von-Guericke University Magdeburg, Magdeburg, Germany; 6 Institute of Public Health, Charité-Universitaetsmedizin Berlin, Corporate Member of Freie Universitaet Berlin and Humbolt- Universitaet zu Berlin, Berlin, Germany; 7 Research Department Climate Resilience, Potsdam Institute for Climate Impact Research (PIK), Member of the Leibniz Association, Potsdam, Germany; 8 Institute of Microbiology, Infectious Diseases and Immunology, Charité-Universitaetsmedizin Berlin, Corporate Member of Freie Universitaet Berlin and Humbolt-Universitaet zu Berlin, Berlin, Germany; 9 Abteilung III, Technische Hochschule Augsburg, Am Silbermannpark 2, 86161 Augsburg, Germany; 10 Interprofessional Graduate School Integrative Medicin and Health, Health Department, Witten/Herdecke University, Witten, Germany; 11 Department of Social Anthropology, Panteion University, Athens, Greece; 12 Faculty of Medicine, Department of Pediatrics, University of São Paulo, São Paulo, Brazil; 13 Department of Pediatric Oncology and Hematology Charité – Universitaetsmedizin Berlin, Corporate Member of Freie Universitaet Berlin, Humboldt-Universitaet zu Berlin and Berlin Institute of Health, Berlin, Germany

**Keywords:** Nutrition, Fasting, Planetary health, Medical education, Interdisciplinary teaching, Mixed-methods, Teaching evaluation, Curriculum enhancement

## Abstract

Nutrition plays a crucial role in human and planetary health, as prevailing nutritional patterns significantly contribute to the global non-communicable disease pandemic. Moreover, the global food system is inextricably linked to planetary health deterioration. The relevance of nutrition for individual and planetary health is insufficiently addressed in German medical schools. Here, we present a two-week course for medical students in the 8th semester at Charité - Universitätsmedizin Berlin on nutrition and fasting in the context of human and planetary health. The course was developed iteratively along the Kern cycle for medical curricula and underwent evaluation through a mixed-methods design spanning across two consecutive semesters. Assessment involved quantitative questionnaires and qualitative interviews. The main exploratory outcome was the overall module rating. Further outcomes encompassed in-depth evaluations of specific aspects, including content, teaching methods, instructor performance, and subjective experiential aspects. The conceptualisation resulted in a highly participative two-week course of 37.5 h. The main learning objectives aimed at the acquisition of technical competences and personal competencies in four areas: (1) the role of nutrition in health and disease; (2) the exploration of global food systems; (3) communication on subjects of nutrition; (4) culinary medicine. Various interactive didactic methodologies were used. 77.8% (*n* = 27) of the participants rated the course as very good or good. The conviction that nutrition influences health and disease increased over the course (Wilcoxon signed rank test, z = -2.82, *p* = 0.005, *r* = 0.57). In the qualitative interviews (*n* = 8) the course content, its didactical diversity and the positive group experience were appreciated. Our results indicate that it could be worthwhile to further incorporate nutrition curricula in medical education.

**Supplementary Information:**

The online version contains supplementary material available at 10.1186/s12909-024-06596-4.

## Introduction

Nutrition fundamentally influences health and disease. In the past 50 years, a detrimental shift in dietary patterns has led to excessive food consumption with imbalanced nutrient profile, resulting in an escalating burden on population health, healthcare systems and environmental stability [1]. Factors related to poor diet were estimated to contribute to 11 million deaths worldwide in 2017 [2]. In the past two decades, evidence suggesting causal relationships between diet and chronic diseases has been published [3], including cardiovascular disease, diabetes mellitus type 2, mental health disorders and certain forms of cancer [4–7], which together account for 74% of deaths before age 70 worldwide [8]. Fasting as a special nutritional intervention has also been shown to be a promising tool for prevention and therapy [9–13]. Thus, diet holds great potential for improving overall health, mitigating the strain on healthcare systems, and promoting a healthier future for the planet.

Additionally, the prevailing global food system including high amounts of animal-source foods significantly contributes to environmental degradation and acts as a major factor for climate change, creating one third of global anthropogenic greenhouse gas emissions, driving deforestation and biodiversity loss and leading to the pollution of soil, air, and water [14–16]. Human wellbeing is inextricably tied to the wellbeing of the planet’s ecosystems [17]. Promoting healthy and sustainable eating habits within populations is therefore a decisive approach to improve human and planetary health [16, 18].

One operational strategy addressing this issue involves advocating for healthy eating practices through healthcare services. Physicians are generally highly trusted in the general population [19], but they need to possess adequate nutrition-related knowledge, motivational skills and competencies to support patients willing to change their diet.

Nevertheless, nutrition education is insufficiently covered in medical curricula globally. Namely, only 44% of international accreditation and curriculum guidelines include nutrition, as shown by a 2021 review. The authors concluded that consensus standards for the integration of nutrition in the curriculum may be necessary [20]. A systematic review of studies covering students’ nutrition knowledge found critical gaps in nutritional understanding, irrespective of the country, setting or year of education. The authors called for more support and training from the faculties in that regard [21]. A European survey of medical schools revealed that nutrition education averaged 23.7 h, Germany providing 11.3 h during the course of medical studies [22]. Medical students in the United Kingdom expressed that their nutrition education was insufficient. Over 70% of 853 surveyed medical students revealed less than two hours of nutrition training during the course of their medical training [23]. In Germany, the federal representation of medical students released a position paper in 2021 stating that the awareness of the connection between nutrition, environment and health is lacking in medical students. The paper further elaborates that integration of nutrition in medical education is insufficient [24]. Although nutrition has recently been integrated in voluntary modules within the National Competence-based Learning Objectives Catalogue (NKLM 2.0) [25], implementation in faculty teaching is sporadic [26].

In response to this global challenge, an increasing number of nutrition curricula for medical students are being initiated lately. A scoping review of nutrition education interventions from 2023 included 23 interventions from around the world. The review focused on the effect of these interventions on students’ knowledge and confidence to implement the learnings in their practice [27]. The Association for Nutrition lead the joint interprofessional development of an undergraduate nutrition curriculum for medical doctors in the UK [28]. A single online nutrition training significantly improved the knowledge of 80 physician assistant students, their confidence in dietary counselling, and their belief in the potential of dietary changes to reverse chronic conditions, with knowledge gains sustained four weeks post-intervention [29]. Others developed a nutrition course for aspiring doctors using a culinary medicine approach [30]. Culinary medicine combines evidence-based nutrition with cooking food, emphasising practical implementation of healthy meals, and considering the immediate reality of the patients [31]. In Germany, an extra-curricular online series of seven online lectures (10,5 h in total) on nutrition, human and planetary health for medical students and physicians was initiated by students at the university of Cologne and launched in 2020 [32]. Nevertheless, a gap in nutrition training persists in German medical education. To address this gap, a two-week elective course on nutrition and fasting for medical students at Charité – Universitätsmedizin Berlin was launched in 2022. Development, implementation and evaluation processes are presented in this paper.

## Materials and methods

### Framework, participants and learning objectives

This course was designed as a two-week programme at the end of the 8th semester of medical education at Charité - Universitätsmedizin Berlin, encompassing 37.5 h over 10 days, for up to 20 participants. Date and duration of the course as well as the number of participants were dictated by the faculty. The course was part of a series of mandatory elective modules offered at the end of the semester for students from the 8th semester. In Germany, the duration of medical studies is typically 12 semesters, comprising four semesters of preclinical and eight semesters of clinical studies. As such, students of the 8th semester have a thorough understanding of preclinical and clinical contexts. All students of the 8th semester have to complete an elective course at the end of the semester. They can choose from a variety of subjects and usually get admitted to their first or second choice.

The course was structured around four main educational objectives developed by a multidisciplinary team and informed by student’s opinions [33, 34]. These were (1) developing technical competences in understanding the content and scientific foundations of the role of nutrition for human and planetary health; (2) exploring the social, political, ecological, and economic factors influencing our food systems; (3) developing personal competencies in effective communication and motivational aspects of promoting healthy eating; and (4) gaining hands-on experience in culinary medicine [35, 36].

### The developmental process

The development of the course followed the framework of Kern for medical education [37].

1. Problem identification: Clinical and teaching experience of the multidisciplinary development team suggested extensive gaps in medical students’ and physicians’ knowledge and application of nutrition in practice (internal communication). Literature supports this perspective [21, 23, 24].
2. The targeted needs assessment was supported by the current process of updating the general curriculum used by the faculty, based on students’ demand for more preventive medicine (internal communication). In the course’s initial iteration, we formally evaluated and engaged with the students to assess needs and preferences.
3. The learning objectives were informed by the needs analysis in steps 1 and 2, considering the concept of Change Management [38] and underwent refinement based on student feedback.
4. Educational strategies and didactic concepts were chosen to fit the learning objectives. For detailed examples, see Table 1.
5. Up to two tutors, at times assisted by one student of a higher semester, depending on the method used, facilitated the practical implementation of the didactic concepts.
6. Following iterations of the course were informed by quantitative and qualitative evaluation as well as informal student feedback.

**Table 1:** Examples of didactical methodologies applied for specific learning objectives connected to main course contents

Didactic approach	Didactic reasoning	Examples of contents where this approach was used
Personal experience / self-experimentation regarding nutrition	As physicians, participants will often be in the position of counselling others to change their dietary habits. Having experienced such a change will help them understand the challenges of the patients.	Challenges and chances of changes in dietary habits
Private study in small groups	Free choice between five thematic options and private study enables participants to strengthen competence in extracting and assessing relevant information from publications and medial information.	Impacts of nutrition on prevalence, prevention and treatment of chronic diseases
Flipped-classroom presentations by small groups	Participants are encouraged to use creative and interactive methods to present their work to the larger group, such as scenic plays, games, quizzes or different visualization techniques. This helps new information to be processed by different parts of the brain and forms a livelier learning experience for the larger group.	Communicating scientific knowledge on nutrition to peers
Presentation	Classical presentations conveying basic knowledge of the field should give an orientation to start with.	Introduction to the potential of nutrition for human health
Thematic workshops with patients from the clinics	These interactive sessions promote active learning, collaboration and problem solving on delineated topics.	Clinical application of different dietary interventions
Patient interviews	Participants are invited to interview patients on their experience with diet change. The goal is to promote the inclusion of nutrition in medical history taking and medical reasoning.	Clinical application of different dietary interventions
Interactive online quiz	Prior knowledge of participants is activated in this gamified element and gives them the opportunity to revise and playfully expand their knowledge.	Specific health effects of plants and plant components
Open questions &answers with a renowned nutrition expert	Participants are given the opportunity to access expert knowledge including practical experience, critical thinking and diverse perspectives. This format brings opportunities for networking and personalized expert guidance.	Various topics guided by the interest of the participants
Culinary medicine with recipe development and collective cooking	The participants are asked to develop recipes combining their knowledge on health, financial and practical aspects with culinary and cooking skills.	Joint conceptualization and cooking of a lunch menu.
Multimedia learning circuit in small groups	This format addresses specific subjects with various types of resources, allowing for different learning styles. Participants can navigate at their own pace, facilitating comprehension. Participants can take ownership of their learning.	Nutrition and planetary health
Simulation game	The Participants are encouraged to use their prior knowledge to formulate convincing arguments to strengthen their position in the complex socio-economic landscape of the game. By identifying with certain players and the immersion into the game, they learn that it needs more than scientific argumentation to navigate through complex socio-political interrelationships in the global debates around nutrition. By this, participants learn to consider broader social determinants of health in consultation with their patients. They are also invited to think about the responsibility of physicians beyond their clinical practice.	Interdependencies of dietary habits of a population with political and economic factors
Patient-oriented (POL) paper cases	Participants are enabled to implement their acquired knowledge in solving clinical cases and offering patients concrete guidance in nutritional interventions. This makes use of competences regarding knowledge of nutritional interventions as well as competencies in motivational interviewing and in supporting sustainable lifestyle changes.	Application of gathered knowledge to a specific clinical case

### Didactic approaches

Medicine as science and art implies the necessity of developing task- and performance-related skills (competences) as well as acquiring attributes and behaviours underlying people-oriented functions (competencies) [39, 40]. Contrasting the rapid dissemination of accessible information on healthy nutrition with the worldwide development of obesity and metabolic diseases [41] shows that providing information on healthy nutrition is insufficient to induce sustainable behavioural changes. Accordingly, student-centred exploration was prioritized over frontal, tutor- and information-centred teaching, and methods that encouraged students to discover underlying principles and engage in dialogues with peers and instructors were applied [42]. The didactic concepts chosen reflect this objective.

A multidisciplinary group of health professionals including physicians, nutrition scientists, dietitians, a psychologist, a researcher in climate change and health, an experimental researcher, a medical student and a student in nutrition science conceptualized and implemented the course. The work of this task force was actively informed and reviewed by a larger clinical research team at Charité - Universitätsmedizin Berlin with longstanding teaching experience.

The course intended to enhance technical competences and personal competencies as outlined in Table 2, exploring the impact of nutrition on individual health and disease. It further covered communication and motivational strategies, while addressing individuals within their psychological, social and economic context. The course progressed towards a broader perspective, elucidating global ecological, social, political and economic dimensions of nutrition. Elements of culinary medicine were integrated throughout the curriculum. Great value was placed on the interaction between students and tutors. The course prioritized collaborative engagement as a fundamental part of the learning process.

Table 1 displays practical applications of the didactic concepts for the core contents of the course, along with the rationale behind their selection.

**Table 2:** Key components of the course curriculum and learning objectives regarding the development of technical competences and personal competencies

Thematic focus	Technical Competences and Personal Competencies to be acquired
**COMPETENCES**	
Fundamental nutrition concepts	Acquaintance with basic principles of nutrition, including macronutrients, micronutrients, the gut microbiome and their roles in human health
Clinical nutrition	Application of nutritional principles to medical practice, understanding how nutrition impacts various medical conditions and treatment
Nutrition and planetary health	Exploration of the current nutrition system and its sustainability impacts and stimulation for transformative thinking
Dietary guidelines and recommendations	Knowledge of national guidelines, how they inform medical practice and how they influence planetary health
Nutritional assessment and evaluation	Applying methods for assessing and evaluating nutritional status, interventions, and patient outcomes
Practical applications: case studies	Application of theoretical knowledge to case studies
Practical applications: self-experience	Exploration of different nutrition forms and nutrition related interventions in self-experience
Practical applications: shared cooking session	Hands-on experience in the kitchen
**COMPETENCIES**	
Nutrition counselling	Skills development in communicating nutritional information to patients and incorporating dietary advice into medical practice
Interdisciplinary collaboration	Understanding the role of nutrition in collaboration with other healthcare professionals
Self-reflection	Reflecting own dietary and lifestyle habits and the processes of change that enhance a healthy lifestyle, challenges in implementing it, supporting factors and management of relapses

### Evaluation

The course was evaluated in a mixed-methods design in 8th -semester students during the summer semester 2022 (S1) and the winter semester 2022/23 (S2). All participants gave informed consent for participation in the evaluation. Adjustments were made based on evaluation results in the first cycle as well as on tutor availability. The timetable including the changes in S2 and a narrative description of the course are provided in Tables 3a, 3b and 3c (see additional file 1). The evaluation was approved by the ethical committee of the faculty (EA1/125/22) and registered on the German registry for clinical trials (DRKS00031037).

#### Quantitative evaluation

Electronic self-developed questionnaires were answered by participants before and after the course (see additional files 4a and 4b) as well as daily (see additional file 4c). Participants were assigned pseudonyms in form of numerical identifiers for data protection purposes. The primary outcome was the overall assessment of the course on a German school grading scale from 1 to 6 (1 = very good, 2 = good, 3 = satisfactory, 4 = sufficient, 5 = deficient, 6 = insufficient). Secondary quantitative outcomes included preliminary knowledge and attitudes towards nutrition, evaluation of content and learning environment, assessment of self-experience and conviction on the effectiveness of nutrition in managing disease. The end questionnaire evaluated teachings, content, complexity, structure, learning gains, teaching methods, environment and lecturers on continuous or symmetrical Likert scales, based on faculty standard evaluation. It additionally focused on the perception of the importance of nutrition for personal and human health, assessed by continuous or symmetrical Likert scales. The daily questionnaires captured ratings of content, complexity, structure, methods, learnings, environment, and lecturers on continuous or symmetrical scales based on faculty standard evaluation. Further, the students were asked to rate the self-experience, if applicable, on a bidirectional Likert-scale (1 = very positive to 5 = very negative).

##### Data analysis

Data collection was executed on the Limesurvey^®^ platform. The acquired dataset underwent descriptive analysis, facilitated by Microsoft Excel and IBM SPSS Statistics software (Version 29.0.0.0 (241) 2023), and was converted to percentages for visualization. Descriptive statistics were computed to provide a comprehensive overview of the data’s central tendencies (mean) and variability (standard deviation). A within-subjects analysis was conducted using the Wilcoxon signed ranks test for changes in responses before and after the course, given a data distribution that differed from a normal distribution. Effect sizes, confidence intervals, z- and *p*-values were calculated. The significance level used was set at a threshold of *p* ≤ 0.05, in accordance with established conventions. The *p*-values are used for exploratory purposes only, while the effect sizes provide an insight of the importance of the differences. The analysis was conducted as per-protocol analysis to include only those participants who were present and completed the evaluation questionnaires.

The analysis of quantitative data in S1 and S2 are presented in an overview of both semesters together and separately.

#### Qualitative evaluation

Qualitative methods were used to gain a deeper understanding of the individual experience of the students. A phenomenological approach was used to explore how the participants perceived the course, to capture their attitudes towards integrating nutrition in medical education and the perceived barriers. The overall intention was to obtain a more nuanced sense of the students’ opinions and engagement, and therefore a more detailed appraisal of the applicability of the course.

Qualitative data were collected through individual face-to-face interviews. Participant selection occurred according to maximum variation sampling based on observed participation during the course (little, medium and high) to capture diverse perspectives. Experienced qualitative researchers within the research team developed and pretested the interview guide including the interviewees’ experiences, interests, motivation and difficulties during the course (Table 4, see additional file 2). The interviews took place in the participants’ homes, except in one case, where the participant was interviewed at the course location. The interviews were conducted by the author LW and lasted between 30 and 90 min, were recorded and transcribed verbatim with the automatic speech recognition software f4x (Dr Dresing&Pehl GmbH, Marburg, Deutschland), and subsequently checked based on the audio recordings (authors LW, AS). Data analysis was conducted by AS and LW in MAXQDA 2022 (VERBI Software, 2021) using structured-thematic qualitative content analysis [43]. Supervision by an experienced qualitative researcher (author WS) was provided throughout data collection and analysis.

## Results

### The course program

The results of the developmental process, i.e., the detailed course program, is shown in Table 3.

**Table 3:** Final course program including methodological details

Day	Time*	Content	Method	Details
Monday	9 am	Introduction to the course	Buzz groups of two-three participants & interactive exchange in large group Collection of cards on board with expectations and fears Presentation	Warm-up questions like “What is the most disgusting thing you ever ate?” or “What would you change in the canteen menu if you had one option?” are first discussed in buzz groups for two minutes. Few highlights are shared in the larger group. Participants are given cards to write expectations and fears regarding the course. After 5 min they are asked to pin them on the board, expectations and fears are automatically clustered by tutors and clarification of the aims and contents of the course is offered where necessary. Short overview of course schedule
	10.30 am	Introduction to the potential of nutrition for human health	Presentation	General overview on the subject with specific examples of nutrients and their impact mainly on chronic diseases.
	12 pm	Personal experience	A How-To Guide to Self-experimentation regarding nutrition	Short instruction to plant-based nutrition, intermittent fasting and western diet as well as other options participants may want to try during the two weeks of the course.
	12.30 pm	Preparation and group allocation for Tuesday	Participants can choose freely between five thematic options	The participants have the option to select from the following five subjects: impacts of nutrition on prevalence, prevention and treatment of (1) Cardiovascular diseases, (2) Immunological and gastrointestinal diseases, (3) Oncological diseases, (4) Neurological and Psychiatric diseases, (5) Metabolic diseases. The Ps are encouraged to use creative and interactive methods to present their work to the larger group.
Tuesday	9 am	Impacts of nutrition on prevalence, prevention and treatment of chronic diseases	Private study in small groups & preparation of presentations	Materials for each thematic group, including full text scientific publications, lists of links to relevant websites and media, are provided on the online learning platform.
Wednesday	9 am	Impacts of nutrition on prevalence, prevention and treatment of chronic diseases	Flipped-classroom presentations of small groups	Each group is allocated 10 min time for their presentation + 10 min discussion
	11.30 am	Nutrition communication	Multimedia presentation	Theoretical approaches to nutrition counselling (motivational interviewing, motivation building, communication strategies), embedded in practical examples with videos.
Thursday	9 am	Practical implications of dietary changes in chronic diseases	Workshops including interviews with patients	Participants can freely choose one of three workshops. Each workshop offers the opportunity to interview patients: (1) undergoing long-term fasting under medical supervision, (2) having changed their diet to plant-based nutrition, (3) having applied Ayurvedic principles of nutrition to their diet.
	11.30 am	Specific health effects of plants and plant components	Interactive Quiz using the platform Kahoot^®^ [59]	Specific food components such as omega-3 fatty acids, polyphenols, and fibres as well as whole foods such as berries, nuts and different vegetables are addressed in the interactive quiz in which participants can vote on their mobile devices. The quiz uses questions with single- and multiple answer options as well as word clouds, sliders, brainstorming with free text input and slides information nuggets.
Friday	9 am	Practical aspects of nutritional counselling	Open discussion on food records Recipe development of healthy low budget recipes in small groups	Participants are presented with different ways of recording dietary intake and are encouraged to complete a food record for themselves for one day in the upcoming week. Through a stepwise approach participants are confronted with knowledge transfer to concrete recipes that can be used in hospital kitchens or canteens. They are asked to develop a healthy dish, are informed about the usual costs of hospital food and are invited to adapt their recipes accordingly. Then all small groups combine their ideas to form a three-course model menu for the next Friday.
	11.30 am	Questions and answers on health effects of nutrition	Open Questions & Answers with a renowned nutrition expert	Questions for the expert are collected during the previous days by participants and tutors, and asked during this session.
Monday	9 am	Nutrition and Planetary Health**	Introductory Presentation Multimedia learning circuit in small groups Practice oriented group discussions	Introduction into the concepts of one health, planetary health, and the planetary health diet. The presentation depicts the interrelationships between nutrition and greenhouse gas emissions, water use, biodiversity, use of fertilisers, soil quality. Nutrition is embedded in the concept of planetary boundaries [60]. Prepared materials including print materials, videos and audios are offered for four different food related topics These were: (i) nutrition politics, (ii) tipping points and nudging, (iii) solutions for sustainable food production and (iv) solutions on consumer’s side e.g. food in hospitals. Ps are invited to rotate in these rooms in small groups. The proposed time for each room lies at 30 min, but participants can freely choose to take more time for one room or leave subjects out. The participants were divided into four new small groups to discuss solutions to the abovementioned challenges in the context of their future work as physicians (e.g. How would you consult patients on the planetary aspects of their diet? )
Tuesday	9 am	Nutrition communication	Discussions and role plays including KIT-elements (communication, interaction, teamwork)	Practical applications of last weeks’ theoretical frame through discussions and role plays
	11.30 am	Interactions of the human microbiome with health and nutrition	Interactive presentation with short quiz contest between two groups including physical movement	An expert on the human microbiota (author SJ) presented details on the history of the discovery and role of the microbiota, including an overview of the current state of evidence.
	12.30 am	The role of ferments and fermentation for human health	Culinary medicine, station work	Different ferments (milk and water kefir, Kombucha, sour dough, tempeh, sauerkraut, kimchi) were distributed around the large room with matching publications in print and spoons for tastings. Participants were encouraged to take certain ferments home and were provided with directions for their cultivation and recipes.
Wednesday	9 am	Political and economic influences on nutritional habits of a population	Simulation game	Participants are asked to develop a national strategy for healthy nutrition on a population level. In groups of two to three Ps, players such as the media, farmers, politics, industry, health insurance companies, physicians and consumers have three hours to draft a consensus on new policies and guidelines for Germany.
Thursday	9 am	Implementation of the gathered knowledge in specific patient cases	Patient-oriented learning (POL) paper cases	The broad knowledge gathered throughout the course should serve Ps in solving clinical cases and offering patients concrete support and nutritional interventions, taking into account safety considerations as well as psychosocial components. The specifically prepared patient cases unfold stepwise through the questions and suggestions posed in the small groups of Ps solving them.
	10.30 am & 11.30 am	Active breaks	Short games on nutrition including physical activity***	Short games of 10 min - Lists of ingredients of ultra processed foods displayed on different tables around the room must be matched to the respective product names - Relay race with every runner having to write one keyword on plant-based nutrition or fasting on a board at the far end of the room
Friday	9 am	Chances and challenges of changing dietary behaviour	Reflection on participants’ own dietary patterns	Having filled in food records, Ps were asked to share their records and experiences in small groups. Then the discussions on chances and challenges were reflected in the larger group, including reflections of Ps that tried out a new dietary habit in the last two weeks (self-experiments see first day).
	10.30 am	Collective cooking	Culinary medicine	Implementation of the three-course model menu of last Friday.
	12.30 am	Reflection on the course	Open group discussion	Open feedback and reflective discussion, including expectations and fears of the first day.

* Two breaks of 15 min each not separately indicated

**The option of flexible timing was introduced following feedback of Ps after the first course. Some Ps expressed a preference to engage in selected topics at their own pace instead of obtaining a broad overview of all topics. The topic materials were revised after Ps indicated that the content was emotionally burdensome instead of motivating

***Games with physical activity were added during the development of the program, as the Ps showed signs of mental fatigue after studying the POL cases

Abbreviations: KIT = Communication, Interaction and Teamwork. This is a format often used in courses at the Charité-Universitätsmedizin Berlin

### Quantitative results

#### Participants

The courses had 18 and 15 participants in S1 and S2 respectively. Participants’ characteristics, daily attendance and response rates are presented in Table 5.

**Table 5:** Participants’ characteristics, course and survey attendance

Characteristics		S1	S2	*p*	S1 + S2
No. of participants		18	15	/	33
Male: Female		4:14	5:10	0.47**	9:24
Mean age		24.6 (SD 3.9) years	28.2 (SD 5.8) years	0.06*	26.3 (SD 5.1) years
No. of answers basic survey		18	15	/	33
No. of answers end survey		12	13	O.18**	25
Day 1:	attendance	18	15	/	33
	daily survey	0	15	< 0.001**	15
Day 2:	attendance	18	15	/	33
	daily survey	7	15	< 0.001**	22
Day 3:	attendance	18	14	0.27**	32
	daily survey	15	14	0.38**	29
Day 4:	attendance	16	13	0.85**	29
	daily survey	15	13	0.79**	28
Day 5:	attendance	15	15	0.09**	30
	daily survey	12	10	1.00**	23
Day 6:	attendance	17	15	0.35**	32
	daily survey	14	12	0.88**	26
Day 7:	attendance	18	13	0.11**	31
	daily survey	16	11	0.25**	27
Day 8:	attendance	15	14	0.38**	29
	daily survey	13	12	0.60**	25
Day 9:	attendance	15	12	0.80**	27
	daily survey	8	11	0.09**	19
Day 10:	attendance	11	10	0.74**	21
	daily survey	6	4	0.68**	10

S1 = Summer semester 2022; S2 = Winter semester 2022/23; No.=Number; SD = Standard deviation; *Mann-Whitney-U-test; **Pearson-Chi-Square-test

#### Main exploratory outcome

**Fig. 1:**
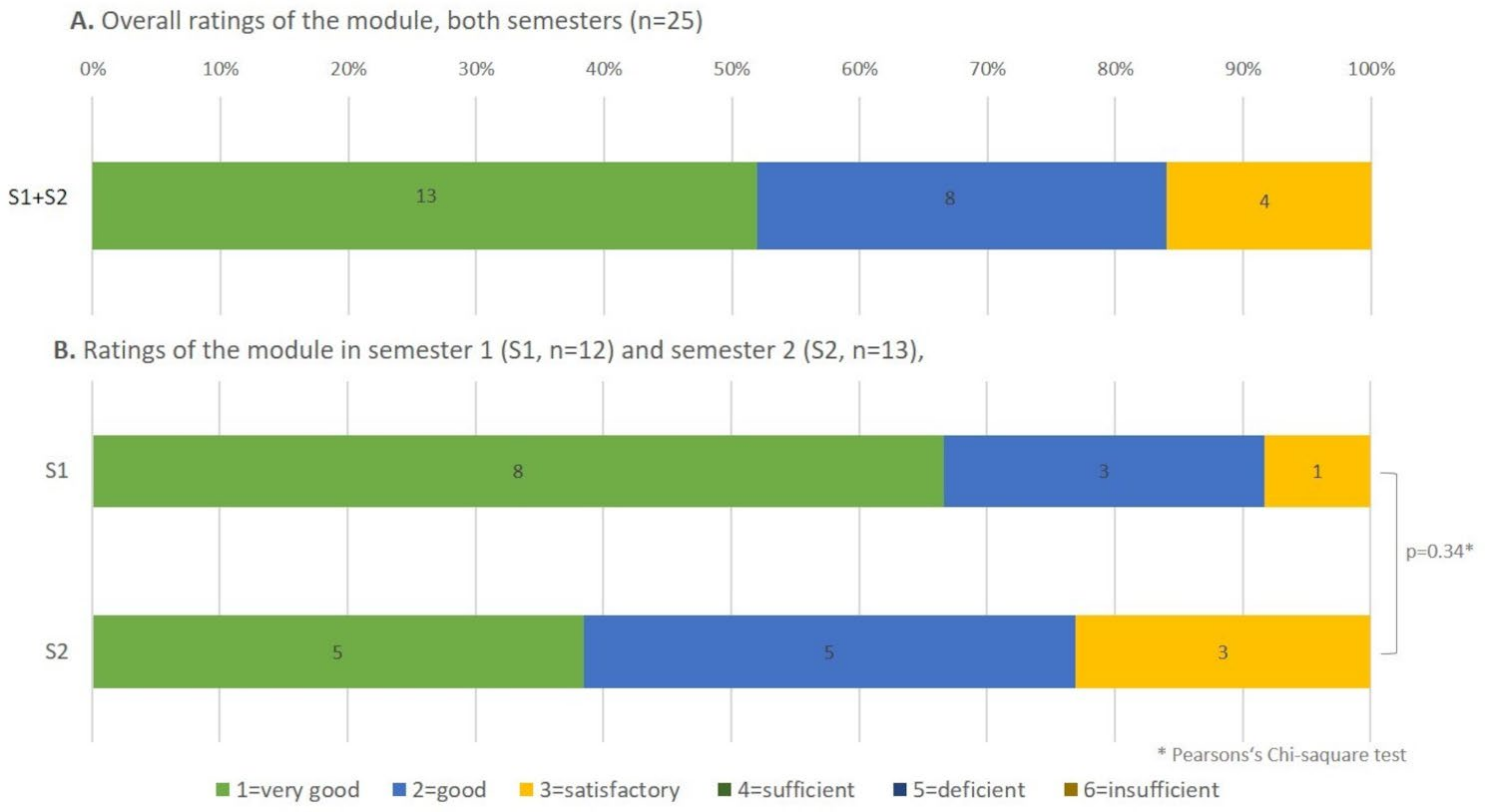
Ratings of the course on a German school grading scale. (**A**) Percentages of ratings in both semesters (S1 + S2) together. (**B**) Percentages of ratings in semester 1 (S1) and semester 2 (S2) separately. S1 = summer semester 2022; S2 = winter semester 2022/2023. The numbers inside the bars represent the number of participants

The main exploratory outcome was the overall evaluation of the module using a German school grading scale. 27 out of 33 students from both courses answered the question “Overall, I give the module the following school grade […]”. 77.8% of total participants rated the module as very good or good. The comparison of the two semesters showed that slightly more participants in S1 rated the course as very good (Fig. 1), although this difference of ratings’ distribution was not significant (Pearsons Chi-Square, *p* = 0.34). When comparing the ratings, no significant differences were detected (Mann-Whitney-U-test, *p* = 0.15).

#### Further outcomes

##### Preliminary knowledge and attitude towards nutrition

Most participants (66.7%, *n* = 33) rated their preliminary nutrition knowledge as good or very good. There was no significant difference between the two semesters (Pearson’s Chi-Square, *p* = 0.35, Mann-Whitney-U-test, *p* = 0.94). Nutrition was described as a relevant topic in individual life by all and in personal environment by 91.0% (*n* = 33) of participants. All participants agreed that physicians should be educated on nutrition for health and disease.

##### Evaluation of content and learning environments

The students were asked to evaluate content and learning environment at the end of each day. In both modules, a minimum of 77.8% of participants rated all course days as “satisfactory” or higher.

**Fig. 2:**
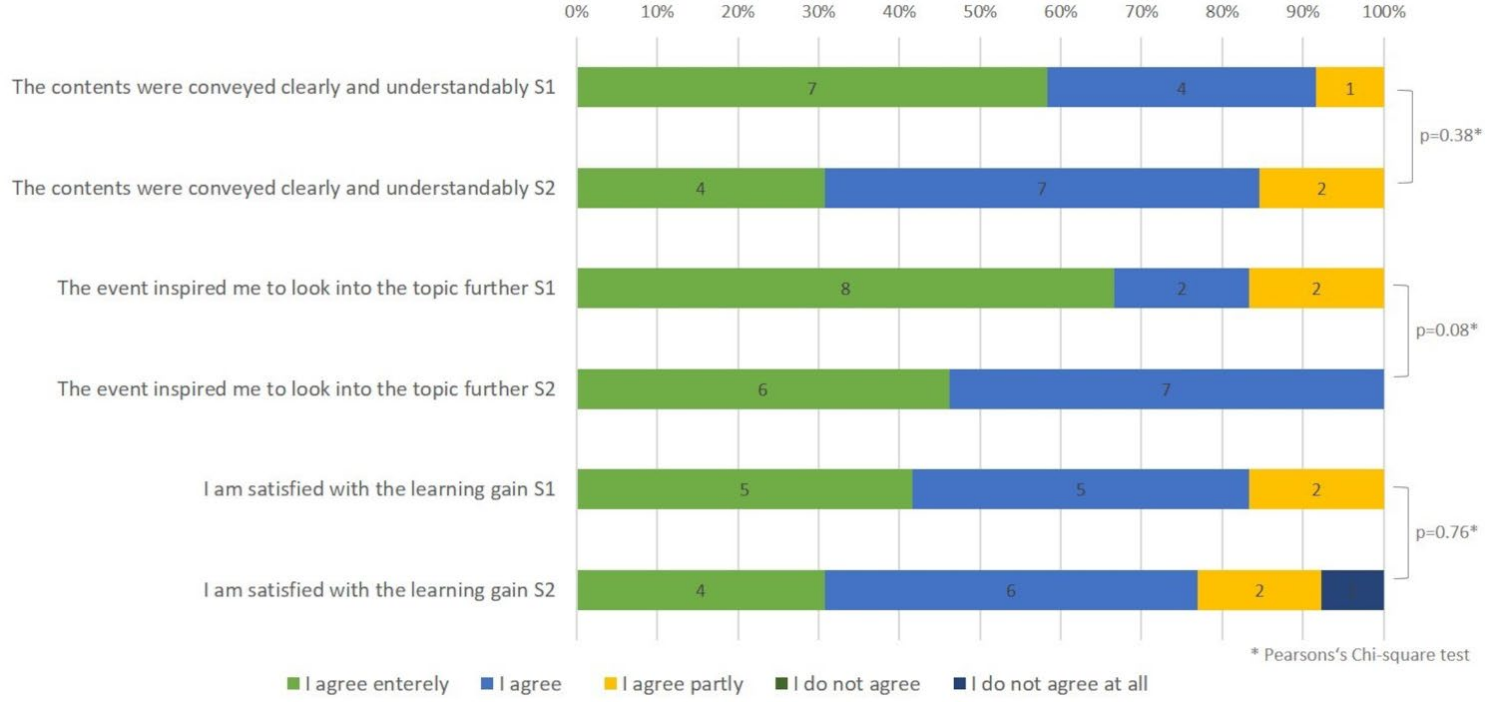
Distribution of participants’ ratings on learning success. Both semesters are shown separately. S1 = summer semester 2022, *n* = 14; S2 = winter semester 2022/2023, *n* = 13. The numbers inside the bars represent the number of participants

Teaching and learning success received predominantly positive ratings, as shown in Fig. 2.

The assessment of the level of difficulty differed between S1 and S2. The module’s content was rated easy by 28.6% in S1- and 61.5% in S2-participants on a bidirectional Likert scale (1 = too easy, 5 = too complex). There was no significant difference between the two semesters (Pearson’s Chi-Square-test, *p* = 0.21, Mann-Whitney-U-test, *p* = 0.07). 81.5% of all participants judged the workload appropriate. The content of the single days was “very well” and “well-coordinated”, according to 88.9% of the students.

The teaching methods were assessed as conducive to learning by 88.9% of participants. The overall use of interactive teaching formats was perceived as appropriate by 92.6% of students.

##### Self-experience

Self-experience was integrated into the module. If interested, participants could choose to fast or to change their diet. 9 out of 18 students in the S1-module and 10 out of 15 in the S2-module tried a dietary self-experience, including switching to a purely plant-based diet or a diet emphasizing on fermented products (the more, the better, up to 6 portions fermented foods a day [44]). These dietary approaches addressed directly (plant-based diets) or indirectly (fasting: moderation and balance; fermented foods: gut and microbiome health) different aspects of the food based dietary guidelines of the German association for nutrition [45, 46]. All the self-experiencing participants deemed the experience of a diet change helpful. 84.2% of the self-experiencing students (*n* = 19) related having a positive experience.

##### Changes of conviction on nutrition

**Fig. 3:**
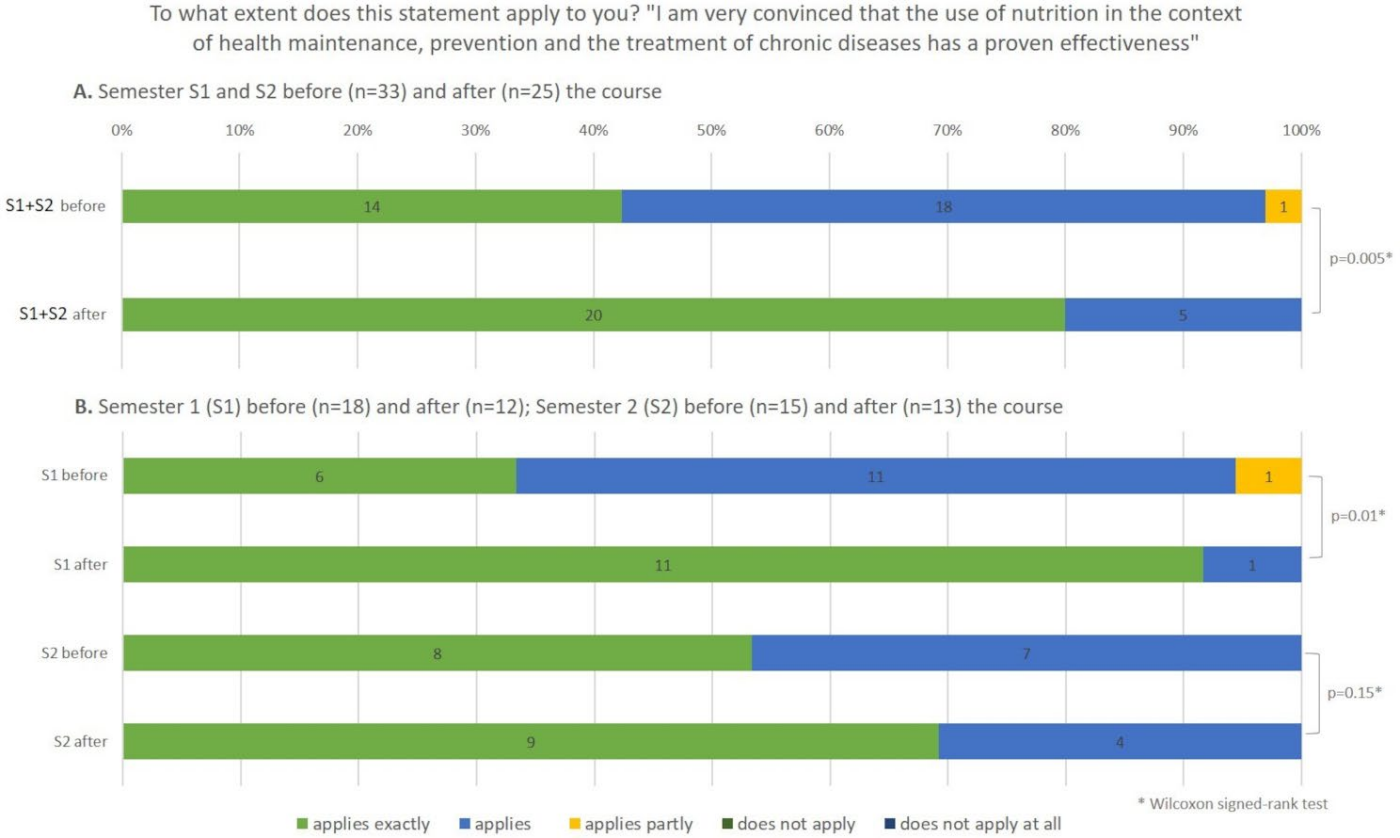
Distribution of ratings on the change of conviction on the role of nutrition in health. Percentage (%) of all participants in both semesters (S1 + S2) (**A**), and separately for semesters S1 and S2 (**B**). The numbers inside the bars represent the number of participants

The students were asked to rate their agreement with the statement, “I am convinced that the use of nutrition in the context of health maintenance, prevention and treatment of chronic disease has a proven effectiveness”. The statement was rated as accurate more often after the module, with a medium effect size, *r* = 0.57 (Wilcoxon-signed-rank-test, *n* = 25, z = -2.83, *p* = 0.005). Looking at the two semesters separately reveals the same trend in both semesters (Fig. 3). In S1, the difference pre-post showed significance (Wilcoxon-signed-rank-test, *n* = 12, z = -2.45, *p* = 0.01, *r* = 0.71), while in S2 the difference was not significant (Wilcoxon-signed-rank-test, *n* = 13, z = -1.41, *p* = 0.15, *r* = 0.39).

### Qualitative results

Qualitative interviews with 10 participants (4 in S1 and 6 in S2) were conducted. Two participants dropped out due to time management issues and unforeseen circumstances. All were native German speakers.

Table 7 (see additional file 3) shows the thematic categories of the structured-thematic qualitative content analysis [43].

The quotes provided were carefully translated from German to English aiming at preserving original meaning, context and tone of the original quote.

#### Motivation for participation

Most participants’ interest was driven by individual and familial motives, complemented by health, self-optimization and performance enhancement. Others stated interest in sustainability and climate change, while others had particular interest in fasting during chemotherapy.

Several interviewees indicated a desire for evidence-based nutrition knowledge and expressed incomprehension regarding the neglect of nutrition in medical education. One student characteristically stated

*[…] Overall, I believe that […] nutrition is covered inadequately in our studies, considering its importance […]” (B2, item 2)*.

Some attendees planned to specialize in general medicine or gastroenterology and wanted to incorporate nutrition into their medical practice. Others expressed a desire to specialize in nutritional medicine and were interested in the experiences of physicians engaged in the field.

Some participants wished to adopt a more comprehensive view of health when dealing with chronic disease, believing that a holistic perspective is crucial for better patient outcomes.

Expectations around learning about scientific evidence and physiological background were reflected by interviewees:

*“[…] in discussions*, *information often seems incomplete and lacking depth. I hoped to get closer to specific solutions.” (B1*, *item 2)*,

while apprehension at the course onset that the content could lack scientific accuracy was also mentioned. Further, participants wished to learn practical nutritional approaches to diseases.

#### Experience of the module

Overall, the course was perceived as well timed and structured coherently, as one interviewee stated,

*“[…] I appreciated the approach of starting from the individual and expanding to the broader perspective. It seemed intentional*, *focusing initially on nutrition for individuals […]. Then*, *transitioning to planetary health […]. Personally*, *it felt coherent and logical*, *making perfect sense*, *the way they structured the course” (B4*, *item 94)*.

However, 4 h daily over the course of 10 days felt insufficient to cover the vast topic of nutrition. The consequence was a perception of crowded schedule in some parts and a superficial approach in others, as expressed by one interviewee:

*“[…] and yes*, *thematically*, *it was incredibly diverse*, *almost overwhelmingly so. Some topics deserved a deeper dive*, *but then the discussion had already shifted to the next item […]” (B1*, *item 50)*.

Other interviewees appreciated the thematic diversity and the reactivity of the course to individual interests.

Overall, the broad variety of evidence-based content and lack of dogmatism were positively reported.

*“[…] But I was all the more surprised by how much evidence was actually presented. How many studies we were given […] and not just personal experiences and feelings. […] And I found that very good*, *and much more strongly represented than I had feared. A sentiment shared by many*, *yes.” (B9*, *item 28).*

The variety of methods was mainly positively assessed:

*“[…] the diversity was exceptional; each day brought something new. We engaged in problem-oriented learning cases one day*, *had lectures the next*, *followed by cooking sessions*, *and a simulation game […]. It was thoroughly enjoyable.” (B1*, *item 50).*

Other participants criticised too much interactive teaching, preferring more frontal lectures by experts:

*“I appreciated the diversity*, *but what I found challenging was the expectation for us to figure everything out on our own. It led me to question: why attend in person if I could accomplish the same tasks at home?” (B8*, *item 46)*.

The commitment of the lecturers was praised, and the module was perceived as designed with passion. The dedication of the students and the rich exchange within the group were also highlighted. One participant stated,

*“[…] I believe everyone who opted for the course was genuinely interested in the subject. In the medical profession*, *there are individuals who lack interest in nutrition […]. Among our group*, *there was a collective eagerness to learn and actively participate. Everyone had valuable insights and opinions to share […]” (B2 II*, *item 24)*.

Many themes were lively and interactively discussed and the positive group atmosphere was consistently emphasised.

#### Highlights and take-home messages

Most attendees described the session on planetary health as one highlight of the course. As a result, participants reported increased awareness and interest in the subject. One participant stated,

*“[…] what struck me the most was looking at my personal impact on the environment through my dietary choices […]. It was a profound realization that deeply resonated with me […]” (B6*, *item 50)*.

Considering the bigger picture and including the whole food system enabled participants to understand interdependencies.

The microbiome was another highlighted topic:

*“I found the topic [of the microbiome] really cool. I personally would have appreciated delving deeper into it […]. The limited understanding we have*, *could easily fill an extended module […]. (B4*, *item 78).*

Some interviewees appreciated the cooking session:

*“Cooking was undoubtedly a highlight for me. […] I was pleasantly surprised by how smoothly the collaborative cooking experience unfolded.” (B4*, *item 90).*

Other mentioned highlights were the expert Q&A, the simulation game, interviewing patients and discovering nutrition counselling.

Collectively, the respondents expressed that the course enabled them to acquire a comprehensive understanding of nutrition rooted in evidence, as the following statement shows:

*“The elective facilitated the engagement with nutrition on a scientific level” (B9*, *item 50).*

Newly acquired knowledge and skills motivated for making novel experiences. Some mentioned starting fermenting foods or experiencing grocery shopping differently.

Some attendees were surprised to realise that minor dietary guidance can result in significant health improvements in patients. One participant related their experience as follows:

*“[…] it [the module] demonstrated to me that as a doctor*, *even small efforts can make a big difference. Initiating a conversation about the topic*, *addressing it briefly*, *and employing techniques like motivational interviewing showed that it is not about making drastic changes over night. […] Providing subtle nudges and small prompts can be enough to instigate meaningful changes in patients.” (B5 item 6).*

Overall, the interviewed participants recognised advantages applicable to future practice, with an even more pronounced impact for their personal health.

#### Lowlights and suggestions

The interviewers specifically asked for unpopular elements in the course. The KIT format (communication, interaction and teamwork) was generally criticised and perceived as overused at Charité Universitätsmedizin Berlin. A few interviewees perceived the traditional Ayurvedic approach to nutrition as irrelevant for physicians. Although the content was described as fascinating, the relationship to medical practice was not always clear to everyone. There was a general desire to discuss more disease patterns in relation to nutrition. Participants mainly wanted more detailed discussions on the role of nutrition in chronic disease, such as cardiovascular disease, metabolic disease, inflammatory disease or oncologic disease, as expressed in informal conversations throughout the course. One interviewee stated:

*“[…] I see it more as a reference material for myself*, *something I can keep in my drawer. These are the dietary guidelines for individuals with diabetes[…] enabling to formulate arguments for the benefit of the patient […]”* (*B7*, *item 40).*

Some interviewees wished for more clarity on learning objectives, as seen in other modules.

Suggestions for improvement included inviting physicians who integrate nutrition into their daily practice and learn from their experience, more interviews with patients who had benefited from nutrition counselling, more preparatory and follow-up work to stretch the busy schedule, a more positive outlook regarding planetary health and real-life examples of how a transformation could work, and expanding the content to cover challenges in nutrition research, deeper microbiome insights, nutrition’s role in ageing and nutrition physiology and psychology.

## Discussion

The two-week course presented here was conceived and implemented as an immersive experience, addressing nutrition in various participative formats.

The developmental process was modelled alongside the process described by Kern et al. [37]. The structural framework of the course (e.g., its duration) was guided by our faculty’s requirements, and while two entire weeks were provided, some participants perceived the course as dense, possibly due to the breadth and complexity of topics discussed. In alignment with the educational goals of the faculty’s practice-oriented medical training, theory was conveyed alongside its clinical applications. The main goal was to give an overview of the importance nutrition holds for health maintenance and disease treatment. Additionally, commonly experienced challenges for patients regarding dietary changes [47] led to a choice of specific subjects like nutrition communication, nutrition environments as well as systemic aspects [46–48]. A critical objective of the course was to incite the conversation on nutrition and planetary health among medical students at the faculty. So far, nutrition has predominantly been addressed in extra-curricular initiatives in medical education in Germany, for example at the universities of Cologne, supported by the Physician Association for Nutrition (PAN) [49], and Munich [50] or initiated by the German Alliance for Climate Change and Health (KLUG) [51]. A pre-clinical nutrition curriculum implemented at Zucker School of Medicine (United States of America) showed increased self-assessed knowledge and comfort in counselling patients in nutrition and a better performance in a nutrition-related examination compared to controls [52, 53]. Unfortunately, in our course, we could only describe direct reactions of the participants to the contents (first level of the Kirkpatrick evaluation model) [54]. The evaluation does, therefore, not properly capture mid- and long-term development of students, assessing learning outcomes, skills and behaviours, and the results of the application of learnings in clinical practice (levels 2–4 of Kirkpatrick model) [54]. Future research should examine the long-term impact of including nutrition-focused education into medical curricula, assessing the effects on graduates’ practices and patient outcomes.

Participants were initially asked about their perception of the knowledge on nutrition amongst physicians. All course participants agreed that physicians needed to be better educated about the importance of nutrition in human health and disease, reflecting findings that aspiring medical professionals recognize the significance of incorporating nutrition counselling and evaluation in clinical practice [46].

The participants welcomed expanding the lens to the broad socio-political context of nutrition. Showing that human and planetary health are fundamentally interdependent was one goal of the course [17]. It was meant to situate nutrition within the broader context of present controversies. One could argue that physicians primarily focus on the individual health of their patients. However, acknowledging the broad environmental and societal context is crucial for understanding potential levers for change. Some scholars argue that teaching formats also have the responsibility of enabling transformative action [55]. The course highlighted the environmental and health impact of individual nutritional choices; one example being the discussion on the benefits of plant-based diets. The students were encouraged to reflect on how their advice to patients could influence personal and environmental health. The course also addressed the food production and transport chain, showing how nutrition is part of a systemic issue. Further, societal and political entanglements of nutrition were explored. Especially the qualitative data reflect how the participants received the information, as connecting nutrition to planetary health was among the most frequently mentioned highlights. Moreover, some attendees expressed a better overall understanding of nutrition; others wanted to improve their dietary habits and be more selective in buying groceries.

Reflecting the interdisciplinarity of the field was another objective of the module. Development and facilitation of the course involved professionals of different disciplines. For instance, nutritionists contributed on dietary guidelines and health effects of specific foods; psychologists and dietitians integrated nutrition psychology and communication; physicians included nutrition in patient care; the environmental scientist addressed ecological aspects and public health experts shared societal and political dimensions. The interdisciplinary focus was well received, as some students pointed out in the interviews, enabling insights from different angles and promoting collaboration. Others pointed out the importance of an interdisciplinary approach to nutrition education. Shared responsibilities across disciplines, including nutritionists, psychologists, doctors, nurses, and public health professionals ensured a comprehensive and all-encompassing patient care. A collaborative approach can further enhance breadth and depth of knowledge as well as practical skills, while fostering communication and teamwork abilities [52, 53, 56, 57].

One limitation of the evaluation of this course was that, given its elective nature, the probability of students who already had a special interest and preliminary knowledge on nutrition participating was high, as they were free to choose the course subject among a variety of elective courses. Nevertheless, differences in background knowledge were revealed in the evaluation as well. The complexity of accommodating different levels of former knowledge, interest and understanding, while still offering useful and practical information to everyone showed itself in a very dense curriculum. We had many interactive elements, resulting in high demands on individual engagement and self-study, which did not satisfy all participants. And although the adherence to the evaluation was high, survey fatigue might have influenced the outcomes. The students’ contributions should have informed the development of the curriculum from the very beginning, as has originally been described by Kern et al. [37]. Future curricula might want to involve more dimensions in their evaluation, following the recommendations of Kirckpatrick et al. [54], which we were not able to implement, due to the short timeframe and elective nature of the course. It might also be helpful for future courses to adapt the validated questionnaire on self-perceived competence of primary health professionals in providing nutrition care to patients with chronic diseases, to a population of medical students [58].

## Conclusion

The course presented here was implemented in a practice-oriented medical curriculum at a large medical university in Germany and was well received by the students, whose feedback informed the course during its development. In particular, the practical elements, the interactive didactical approach and the interdisciplinary teaching were widely appreciated. Future curricula would be well advised to engage with students at an early stage, even before the implementation of the pilot course. This would facilitate students’ involvement in the development of the curriculum, including areas deemed important to them. Overall, providing a new generation of healthcare professionals with the awareness of the transformative potential of nutrition and the skills to accompany lifestyle changes in daily practice, could be part of a solution for the global human and planetary health challenges humanity is currently facing.

## Electronic supplementary material

Below is the link to the electronic supplementary material.

Additional File 1: Ngoumou-Koppold_BMC-Medical-Education. Table 3: 3a: Timetable of the course in the sommer semester 2022 (S1); 3b: Modifications of the timetable in the winter semester 2022/23 (S2). Table 3a and 3b show the changes made to the timetable for the second evaluation cycle

Additional File 2: Ngoumou-Koppold_BMC-Medical-Education. Table 4: Interview guide for the qualitative course evaluation interviews. Table 4 shows the structured set of questions used to guide through the qualitative evaluation interviews

Additional File 3: Ngoumou-Koppold_BMC-Medical-Education. Table 7: Qualitative thematic category system. Table 7 shows the code system deduced from the qualitative interviews, within which the content of the interviews have been categorised for analysis

Additional File 4a: Ngoumou-Koppold_BMC-Medical-Education. Quantitative self-developed Baseline questionnaire, delivered before the course

Additional File 4b: Ngoumou-Koppold_BMC-Medical-Education. Quantitative self-developed final questionnaire, delivered after the course

Additional File 4c: Ngoumou-Koppold_BMC-Medical-Education. Quantitative self-developed daily questionnaire, delivered daily

## Data Availability

The datasets used and/or analysed during the current study are available from the corresponding author on reasonable request.

## Declarations

### Study registration

The study was registered on the German Registry for clinical Trials (DRKS00031037).

## Abbreviations

KIT: Communication, Interaction and Teamwork
KLUG: German Alliance for Climate Change and Health
NKLM: National Competency-Based Learning Objectives Catalogue for Medicine
PAN: Physician Association for Nutrition
POL: Problem Oriented Learning
Ps: Participants
S1: Summer semester 2022
S2: Winter semester 2022/2023
