# Supplementary material for: An interactive course program on nutrition for medical students: interdisciplinary development and mixed-methods evaluation
Source: BMC Med Educ. 2025 Jan 23;25:115. doi: 10.1186/s12909-024-06596-4 (PMC11761204; doi:10.1186/s12909-024-06596-4)
Supplement: Supplementary file 1 — Additional File 1: Ngoumou-Koppold_BMC-Medical-Education. Table 3: 3a: Timetable of the course in the sommer semester 2022 (S1); 3b: Modifications of the timetable in the winter semester 2022/23 (S2). Table 3a and 3b show the changes made to the timetable for the second evaluation cycle [file 12909_2024_6596_MOESM1_ESM.docx]

**Ngoumou & Koppold et al. A Transformative Nutrition Course for Medical Students: Interdisciplinary Development and Mixed-Methods Evaluation. Manuscript submitted at BMC Medical Education.**

**Additional file 2** – Table 4

**Table 4.** Interview guide for the qualitative course evaluation interviews

| The course   - Motivation to take part in the course, expectations and wishes of the course. - What did you particularly like? What did you not like? Why? - Which events did you find particularly informative? Illustrative? Interesting? And why? - What did you learn? What is your personal take-home message? - How did you like the teaching methods used? What was good and what was bad? Please get specific. - What did you miss? What would you change? What do you wish for?   Nutrition in general   - Attitude towards nutrition in personal environment and in the context of planetary health before and after the elective   Nutrition in medical studies and in medicine   - Attitude towards the evidence of the role of nutrition in health maintenance, prevention and treatment of chronic diseases - Opinion on the status of nutrition in medical studies, what should change? |
| --- |
