# Supplementary material for: An interactive course program on nutrition for medical students: interdisciplinary development and mixed-methods evaluation
Source: BMC Med Educ. 2025 Jan 23;25:115. doi: 10.1186/s12909-024-06596-4 (PMC11761204; doi:10.1186/s12909-024-06596-4)
Supplement: Supplementary file 2 — Additional File 2: Ngoumou-Koppold_BMC-Medical-Education. Table 4: Interview guide for the qualitative course evaluation interviews. Table 4 shows the structured set of questions used to guide through the qualitative evaluation interviews [file 12909_2024_6596_MOESM2_ESM.docx]

**Ngoumou & Koppold et al. A Transformative Nutrition Course for Medical Students: Interdisciplinary Development and Mixed-Methods Evaluation. Manuscript submitted at BMC Medical Education.**

**Additional file 3** – Table 7

**Table 7.** Qualitative thematic category system

| *Main code* | *Code* | *Subcode* |
| --- | --- | --- |
| *1 Motivation for participation in the course* | 1.1. Reasons for participation  1.2. Expectations | 1.1.1. (Personal) interest in nutrition  1.1.2. Desire for scientific knowledge  1.1.3. Importance of nutrition for work as physicians  1.1.4. Wish for holistic view on health |
| *2 Experience of the course* | 2.1. Structure of the course  2.2. Content of the course  2.3. Methods and formats used  2.4. Lecturers  2.5. Atmosphere |  |
| *3 Perceived highlights* | 3.1. Related to the methods  3.2. Related to the content  3.3. Other |  |
| *4 Take-home messages* | 4.1. Overall take-home messages  4.2. Professional take-home messages  4.3. Personal take-home messages |  |
| *5 Perceived lowlights* | 5.1. Related to the methods  5.2. Related to the content  5.3. Other |  |
| *6 Suggestions for improvement* | 6.1. Related to the content  6.2. Related to the methods  6.3. Related to the overall structure |  |
