# Supplementary material for: An interactive course program on nutrition for medical students: interdisciplinary development and mixed-methods evaluation
Source: BMC Med Educ. 2025 Jan 23;25:115. doi: 10.1186/s12909-024-06596-4 (PMC11761204; doi:10.1186/s12909-024-06596-4)
Supplement: Supplementary file 3 — Additional File 3: Ngoumou-Koppold_BMC-Medical-Education. Table 7: Qualitative thematic category system. Table 7 shows the code system deduced from the qualitative interviews, within which the content of the interviews have been categorised for analysis [file 12909_2024_6596_MOESM3_ESM.docx]

**Final Survey**

1. **What is your participant number?**

2. **The content of the module was for my level of knowledge...**

- 1 Too easy

- 2 Easy

- 3 Just right

- 4 Complex

- 5 Too complex

3. **The workload in this module made me feel...**

- 1 Very under challenged

- 2 under challenged

- 3 Neither over- nor under challenged

- 4 over challenged

- 5 Very over challenged

4. **The content of the individual module events was...**

- 1 Very well coordinated

- 2 Well coordinated

- 3 Partially well coordinated

- 4 Not well coordinated

- 5 uncoordinated

5. **I am …….. with the learning gain from this module**

- 1 Very satisfied

- 2 Satisfied

- 3 Partially satisfied

- 4 Not satisfied

- 5 Not satisfied at all

6. **How do you rate the incorporation of self-experience into the course?**

- Very useful

- Useful

- Partially useful

- Useless

- Very useless

7. **The course was well organised**

- 1 Strongly agree

- 2 Agree

- 3 Neutral

- 4 Disagree

- 5 Strongly disagree

8. **The level of difficulty was...**

- 1 Much too high

- 2 Too high

- 3 Appropriate

- 4 Too low

- 5 Much too low

9. **The amount of learning objectives was...**

- 1 Much too much

- 2 Too much

- 3 Appropriate

- 4 Too little

- 5 Much too little

10. **The methods used were conducive to learning**

- 1 Strongly agree

- 2 Agree

- 3 Neutral

- 4 Disagree

- 5 Strongly disagree

11. **In video and audio recordings, the teachers appeared engaged**

- 1 Strongly agree

- 2 Agree

- 3 Neutral

- 4 Disagree

- 5 Strongly disagree

12. **There were technical problems using the platform**

- Yes

- No

13. **The use of interactive learning formats such as exercises, quizzes, MC questions, Kahoot, etc., was...**

- 1 Too little

- 2 Little

- 3 Appropriate

- 4 Much

- 5 Too much

14. **The use of recordings was...**

- 1 Too little

- 2 Little

- 3 Appropriate

- 4 Much

- 5 Too much

15. **The use of videos was...**

- 1 Too little

- 2 Little

- 3 Appropriate

- 4 Much

- 5 Too much

16. **My workload for this course was...**

- Too little

- Little

- Appropriate

- Much

- Too much

17. **The content was conveyed clearly and understandably**

- 1 Strongly agree

- 2 Agree

- 3 Neutral

- 4 Disagree

- 5 Strongly disagree

18. **The course stimulated me to further engage with the topic**

- 1 Strongly agree

- 2 Agree

- 3 Neutral

- 4 Disagree

- 5 Strongly disagree

19. **I am satisfied with the learning gain**

- 1 Very satisfied

- 2 Satisfied

- 3 Partially satisfied

- 4 Dissatisfied

- 5 Very dissatisfied

20. **Did you undertake a self-experience as part of the elective internship?**

- Yes

- No

21. **How meaningful do you find the integration of self-experience into the elective internship?**

- Very useful

- Useful

- Neutral

- Useless

- Very useless

22. **How did you experience the self-experience?**

- Very positive

- Positive

- Neutral

- Negative

- Very negative

23. **Do you have any comments regarding the self-experience?**

(Ideas, suggestions, change proposals, notes, etc.)

24. **To what extent do you agree with the following statement: I am strongly convinced that the use of nutrition in the context of health maintenance, prevention, and treatment of chronic diseases has proven effectiveness.**

- Strongly agree

- Agree

- Neutral

- Disagree

- Strongly disagree

25. **I give the course the following grade overall...**

- 1 Very good

- 2 Good

- 3 Satisfactory

- 4 Adequate

- 5 Poor

- 6 Inadequate

26. **Overall, my expectations were...**

- Completely fulfilled

- Fulfilled

- Neutral

- Not fulfilled

- Not fulfilled at all

27. **Do you have any comments regarding the course overall?**

(Notes, wishes, ideas, tips, etc.)
