# Supplementary material for: An interactive course program on nutrition for medical students: interdisciplinary development and mixed-methods evaluation
Source: BMC Med Educ. 2025 Jan 23;25:115. doi: 10.1186/s12909-024-06596-4 (PMC11761204; doi:10.1186/s12909-024-06596-4)
Supplement: Supplementary file 4 — Additional File 4a: Ngoumou-Koppold_BMC-Medical-Education. Quantitative self-developed Baseline questionnaire, delivered before the course [file 12909_2024_6596_MOESM4_ESM.docx]

**Baseline Survey**

1. **What is your participant number?**

2. **Please state your age**

3. **Please state your gender**

- Male

- Female

- Diverse

- No answer

4. **What would you like to try in the two weeks of self-experience?**

- Intermittent fasting

- Plant-based diet

- Fast food only

- Other

5. **How would you rate your knowledge about nutrition?**

- 1 Very good

- 2 Good

- 3 Average

- 4 Poor

- 5 Very poor

6. **The topic of nutrition is personally relevant to me**

- 1 Strongly agree

- 2 Agree

- 3 Neutral

- 4 Disagree

- 5 Strongly disagree

7. **The topic of nutrition is relevant in my private environment**

- 1 Strongly agree

- 2 Agree

- 3 Neutral

- 4 Disagree

- 5 Strongly disagree

8. **Nutrition plays an important role in maintaining health**

- 1 Strongly agree

- 2 Agree

- 3 Neutral

- 4 Disagree

- 5 Strongly disagree

9. **Nutrition plays an important role in disease prevention**

- 1 Strongly agree

- 2 Agree

- 3 Neutral

- 4 Disagree

- 5 Strongly disagree

10. **Nutrition plays an important role in the treatment of chronic diseases**

- 1 Strongly agree

- 2 Agree

- 3 Neutral

- 4 Disagree

- 5 Strongly disagree

11. **There is good scientific evidence on the role of nutrition in maintaining health**

- 1 Strongly agree

- 2 Agree

- 3 Neutral

- 4 Disagree

- 5 Strongly disagree

12. **There is good scientific evidence on the role of nutrition in disease prevention**

- 1 Strongly agree

- 2 Agree

- 3 Neutral

- 4 Disagree

- 5 Strongly disagree

13. **There is good scientific evidence on the role of nutrition in the treatment of chronic diseases**

- 1 Strongly agree

- 2 Agree

- 3 Neutral

- 4 Disagree

- 5 Strongly disagree

14. **Doctors should be aware of the role of nutrition**

- 1 Strongly agree

- 2 Agree

- 3 Neutral

- 4 Disagree

- 5 Strongly disagree

15. **Have you already gained fasting experience?**

- Yes, once

- Yes, multiple times

- No

16. **If yes, what type of fasting have you already practiced?**

Please choose the applicable points and comment:

- Multi-day therapeutic fasting (Comment: maximum fasting duration)

- Religious fasting

- Intermittent fasting (e.g., one day a week or several hours a day)

- Other (enter in the comment field)

17. **If you practice therapeutic fasting, how often do you fast?**

- Less than once a year

- 1-2 times a year

- 3-5 times a year

- 6-9 times a year

- More than 10 times a year

18. **To what extent do you agree with the following statement: I am strongly convinced that the use of nutrition in the context of health maintenance, prevention, and treatment of chronic diseases has proven effectiveness.**

- Completely true

- Mostly true

- Neutral

- Mostly not true

- Not true at all
