# Supplementary material for: An interactive course program on nutrition for medical students: interdisciplinary development and mixed-methods evaluation
Source: BMC Med Educ. 2025 Jan 23;25:115. doi: 10.1186/s12909-024-06596-4 (PMC11761204; doi:10.1186/s12909-024-06596-4)
Supplement: Supplementary file 5 — Additional File 4b: Ngoumou-Koppold_BMC-Medical-Education. Quantitative self-developed final questionnaire, delivered after the course [file 12909_2024_6596_MOESM5_ESM.docx]

**Daily Survey**

1. **What is your participant number?**

2. **The tutors were able to convey the content well**

- 1 Strongly agree

- 2 Agree

- 3 Neutral

- 4 Disagree

- 5 Strongly disagree

3. **The learning content was appropriate for the time available**

- 1 Strongly agree

- 2 Agree

- 3 Neutral

- 4 Disagree

- 5 Strongly disagree

4. **The course day was didactically well structured**

- 1 Strongly agree

- 2 Agree

- 3 Neutral

- 4 Disagree

- 5 Strongly disagree

5. **The course day encouraged participation and further learning**

- 1 Strongly agree

- 2 Agree

- 3 Neutral

- 4 Disagree

- 5 Strongly disagree

6. **I am satisfied with my learning gain**

- 1 Very satisfied

- 2 Satisfied

- 3 Neutral

- 4 Dissatisfied

- 5 Very dissatisfied

7. **Overall, I give the course today the following grade...**

- 1 Very good

- 2 Good

- 3 Satisfactory

- 4 Adequate

- 5 Poor

- 6 Inadequate

8. **The course day was well organized**

- 1 Strongly agree

- 2 Agree

- 3 Neutral

- 4 Disagree

- 5 Strongly disagree

9. **The level of difficulty was...**

- 1 Much too high

- 2 Too high

- 3 Appropriate

- 4 Too low

- 5 Much too low

10. **The amount of learning objectives was...**

- 1 Much too much

- 2 Too much

- 3 Appropriate

- 4 Too little

- 5 Much too little

11. **The methods used were conducive to learning**

- 1 Strongly agree

- 2 Agree

- 3 Neutral

- 4 Disagree

- 5 Strongly disagree

12. **In video and audio recordings, the teachers appeared engaged**

- 1 Strongly agree

- 2 Agree

- 3 Neutral

- 4 Disagree

- 5 Strongly disagree

13. **There were technical problems using the platform**

- Yes

- No

14. **The use of exercises, quizzes, MC questions, Kahoot, etc., was...**

- 1 Too little

- 2 Little

- 3 Appropriate

- 4 Much

- 5 Too much

15. **The use of recordings was...**

- 1 Too little

- 2 Little

- 3 Appropriate

- 4 Much

- 5 Too much

16. **The use of videos was...**

- 1 Too little

- 2 Little

- 3 Appropriate

- 4 Much

- 5 Too much

17. **My workload was...**

- 1 Very low

- 2 Low

- 3 Appropriate

- 4 High

- 5 Very high

18. **The content was conveyed clearly and understandably**

- 1 Strongly agree

- 2 Agree

- 3 Neutral

- 4 Disagree

- 5 Strongly disagree

19. **The course stimulated me to further engage with the topic**

- 1 Strongly agree

- 2 Agree

- 3 Neutral

- 4 Disagree

- 5 Strongly disagree

21. **Overall, I am satisfied with the course day**

- 1 Very satisfied

- 2 Satisfied

- 3 Neutral

- 4 Dissatisfied

- 5 Very dissatisfied
