# Supplementary material for: An interactive course program on nutrition for medical students: interdisciplinary development and mixed-methods evaluation
Source: BMC Med Educ. 2025 Jan 23;25:115. doi: 10.1186/s12909-024-06596-4 (PMC11761204; doi:10.1186/s12909-024-06596-4)
Supplement: Supplementary file 6 — Additional File 4c: Ngoumou-Koppold_BMC-Medical-Education. Quantitative self-developed daily questionnaire, delivered daily [file 12909_2024_6596_MOESM6_ESM.docx]

**Ngoumou & Koppold et al. A Transformative Nutrition Course for Medical Students: Interdisciplinary Development and Mixed-Methods Evaluation. Manuscript submitted at BMC Medical Education.**

**Additional file 1 -** Tables 3a and 3b

**Table 3a.** Timetable of the course in the summer semester 2022 (S1)

| **Day 1**  9:00-13:00 | **Day2**  9:00-13:00 | **Day 3**  9:00-13:00 | **Day 4**  9:00-13:00 | **Day 5**  9:00-13:00 | **Day 6**  9:00-13:00 | **Day 7**  9:00-13:00 | **Day 8**  9:00-13:00 | **Day 9**  9:00-13:00 | **Day10**  9:00-13:00 |
| --- | --- | --- | --- | --- | --- | --- | --- | --- | --- |
| Getting to know each other, exploring motivations and apprehensions  (Clinician researcher) | Preparation for the upcoming presentations in groups on day 3. 5 groups working on 5 disease groups (i) cardiovascular (ii) metabolic (iii) oncologic (iv) inflammatory (v) central nervous system  Resources are provided on the online learning platform  *No on-site attendance is necessary* | Group presentations  20-25 minutes per group, incl. discussion.  (Students, moderated by clinician researcher) | Nutrition as therapy: 3 parallel workshops, incl. patient presentations  -Fasting (Clinician researcher)  -Ayurveda (Dietitian)  -Plant-based nutrition (Clinician researcher) | Menu discussion for shared cooking session on day 10  (Dietitian) | Topic planetary health and nutrition  Lecture, station work, group tasks  (Research physician on planetary health) | Three POL^a^ cases based on the workshops  (Clinician researcher, student) | Simulation game  "Germany seeks a food strategy"  (Clinician researcher, student) | Communication on topics regarding nutrition with lecture, practical examples, KIT^b^-session  (Psychologist, nutritionist, dietitian) | Evaluation of food protocols  (Dietitian) |
| *Video lecture*: General overview of nutrition and health  (Clinician researcher) |  |  |  |  |  |  |  |  | Evaluation of self-experience  (Dietitian, clinician researcher) |
|  |  |  |  | Nutrition protocol, nutrient calculation  (Dietitian) |  |  |  |  |  |
|  |  |  |  |  |  |  |  |  | Cooking together  (Everyone) |
|  |  |  |  | Reflection on self-experience  (Dietitian, clinician researcher) |  |  |  |  |  |
| *Lecture:* Nutrition and research  (Clinician researcher) |  |  |  |  |  |  |  |  |  |
|  |  | *Lecture:* Introduction to the microbiome and  fermented food tasting  (Microbiome researcher) | How our diet contributes to treatment and prevention: a learning-game on a game-based learning platform  (Dietitian) |  |  |  |  |  |  |
| Introduction to voluntary self-experience |  |  |  |  |  |  |  |  |  |
|  |  |  |  |  |  |  | Discussion |  | Buffet lunch &  Q&A time  (Everyone) |
|  |  |  |  | Expert Q&A  (Clinician Researcher) |  |  |  |  |  |
| *Presenting the timetable and explaining group task* |  |  |  |  |  | Preparation for simulation game |  |  |  |

^a^ POL=Problem Oriented Learning, ^b^ KIT= Communication, Interaction, Teamwork, Q&A=Questions and answers

**Table 3b.** Modifications of the timetable in the winter semester 2022/23 (S2)

| **Day 1** | **Day 2** | **Day 3** | **Day 4** | **Day 5** | **Day 6** | **Day 7** | **Day 8** | **Day 9** | **Day 10** |
| --- | --- | --- | --- | --- | --- | --- | --- | --- | --- |
| No lecture on Nutrition and Research | No changes | Introduction to nutrition communication instead of the Microbiome lecture | No changes | No expert Q&A | No changes | Nutrition communication – deepening  Lecture: Introduction to the microbiome and fermented food tasting | No changes | POL cases based on workshops  Expert Q&A | No changes in content, one change in tutor, change in location |
| Same tutors | Same tutors | One tutor for the topic of communication on nutrition changed | Same tutors | One Tutor changed | Same tutors | One tutor for the topic of communication on nutrition changed | Same tutors | Same tutors | One tutor changed |

**Table 3c.** Narrative description of the course

| **Day** | **Narrative description** |
| --- | --- |
| **Day 1** | The course begins with an introductory session. Participants get to know each other and the tutors and are asked to share their motivations and concerns, which intends to create a sense of community. The introductory session is followed by a video lecture on nutrition and human health, after which the content is jointly discussed. Then, a short overview of the challenges in clinical research on nutrition is offered and subsequently discussed. The participants are then introduced to the idea of self-experience and encouraged to implement dietary changes during the course. The day concludes with explanations on the group tasks for the next day, and participants are divided into 5 groups. |
| **Day 2** | Students work on allocated topics in small groups focusing on nutrition in the context of different diseases. The 5 topics are nutrition in cardiovascular risk factors and disease, in metabolic risk factors and disease, in oncologic disease, in inflammatory and gastro-enterological disease, and in neurological and psychiatric disease. Recent scientific resources are provided via an online platform, and students are encouraged to look for further resources by themselves. Students are encouraged to create engaging presentations for their peers for day 3. No on-site presence is required on that day, the small groups can work where ever they wish. |
| **Day 3** | Each group presents their findings, followed by a discussion. During the second part of the day, an interactive introductive lecture to the human microbiome is presented. The students are then introduced to fermented foods and their health benefits, combined with a tasting of several common fermented foods, connecting theory and practice. |
| **Day 4** | The focus shifts to nutrition as therapy, and students are introduced to 3 different dietary approaches (fasting, Ayurveda-nutrition, plant-based-nutrition) via workshops in small groups. Then, students learn about evidence-based effects of single foods and food combinations in a gamified interactive presentation. |
| **Day 5** | In an interactive process, students collaboratively design a 3-course menu for the shared cooking session on day 10, considering different aspects such as health, sustainability and budget (a. o.). The students collaboratively determine the cost of the menu and discussed how much each person is willing to contribute. Important tasks (buying ingredients, cooking, cleaning, etc) are distributed. The session intends to make students think critically about how nutrition, health and social and financial aspects may be related. They are also introduced to food protocols and nutrient calculation. The day closes with a Q&A session with a nutrition expert. |
| **Day 6** | This day is dedicated to the subject of planetary health and nutrition. A lecture informs on the current evidence-based knowledge on the topic. Station work on specific sub-topics (food production, biodiversity, tipping points, role of politics, food in hospitals) enable to delve deeper into some aspects of the subject. In group tasks, students explore the intersection of nutrition and environmental sustainability and the specific role of health-professionals. Scientific resources for further read are provided on the online platform. |
| **Day 7** | Students engage in problem-oriented learning (POL) through three clinical cases based on the workshops on day 4, fostering practical application of their learnings. Students work in 3 small groups, mixing participants of the 3 workshops. |
| **Day 8** | A self-developed simulation game entitled ‘Germany seeks a food strategy immerses students in policy-making, emphasizing the role of nutrition in national strategies. Students are encouraged to reflect on the larger role health professionals and other stakeholders play in the development of dietary guidelines. |
| **Day 9** | Nutrition communication skills are explored through lectures, practical examples, and KIT (communication, interaction, teamwork) sessions, where students practice communication methods. This day focuses on patient interaction and effective communication. |
| **Day 10** | The last day is dedicated to a shared cooking session. Alongside the cooking process, health benefits of individual ingredients and food combinations are explored. The course concludes with a buffet lunch, bringing students and tutors together at the lunch table. |
